# Identification of a novel *KCNT2* variant in a family with developmental and epileptic encephalopathies: a case report and literature review

**DOI:** 10.3389/fgene.2024.1371282

**Published:** 2024-03-06

**Authors:** Fengji Cui, Tuoya Wulan, Qian Zhang, Victor Wei Zhang, Yuhua Jiang

**Affiliations:** ^1^ Department of Molecular Genetics, Chifeng Maternity Hospital, Chifeng, China; ^2^ Department of Reproduction, Chifeng Maternity Hospital, Chifeng, China; ^3^ AmCare Genomics Lab, Guangzhou, China; ^4^ Department of Obstetrics, Chifeng Maternity Hospital, Chifeng, China

**Keywords:** DEE, epilepsy, intellectual disability, KCNT2, KATP channel

## Abstract

**Background:** Developmental and epileptic encephalopathies (DEEs) are a group of heterogeneous neurodevelopmental diseases characterized mainly by developmental delay/intellectual disability and early-onset epilepsy. Researchers have identified variations in the *KCNT2* gene (OMIM* 610044) as the cause of DEE type 57 (MIM# 617771).

**Case presentation:** We report in this study a 46-year-old woman who presented with early-onset epilepsy, intellectual disability, hypertrichosis, coarse facial features, and short stature. Besides, there were four other affected individuals in her family history, including two elder brothers, a younger brother, and their mother. We collected blood samples from the proband, her two affected brothers, and her clinically normal daughter for genetic analysis. Clinical exome sequencing revealed a novel heterozygous variant in the *KCNT2* gene (NM_198503: c.188G>A, p.Arg63His) in the proband and her two affected brothers, while her daughter did not carry this variant. Furthermore, we reviewed all 25 patients identified in the literature with *KCNT2* variants and compared their phenotypes.

**Conclusion:** Epilepsy and intellectual disability/developmental delay occur in almost all patients with *KCNT2* variants. *KCNT2*-relevant DEEs partially overlap with the clinical phenotypes of K_ATP_ channel diseases, particularly in hypertrichosis and distinctive coarse facial features.

## Introduction

“Developmental and epileptic encephalopathy” (DEE), as defined by the International League Against Epilepsy (ILAE), refers to disorders influenced by developmental factors. These disorders frequently exhibit epileptic activity, significantly impacting brain development and functional capabilities (Scheffer et al., 2016; Scheffer et al., 2017). DEEs, characterized by their early onset and severity, often lead to long-term developmental and cognitive impairments. They are heterogeneous, with a high genetic etiology rate, and are among the most severe forms of epilepsy. DEEs typically appear in infancy or early childhood, with many cases presenting within the first year of life (Guerrini et al., 2023). Although each disease is relatively rare on an individual level, collectively, the overall incidence rate of DEE is about 1 in 340 children. Specifically, 1 in 590 children suffer from developmental and epileptic encephalopathy (DEE), and 1 in 800 children have both intellectual disability and epilepsy (ID + E) (Poke et al., 2023). DEEs severely affect the affected children’s quality of life and impose significant burdens on families and society (Palmer et al., 2021).

Numerous genes linked to epileptic encephalopathies are also involved in developmental impairments, indicating their dual role in these disorders. Thus, the underlying genetic cause may result in developmental delay (DD) and/or ID in its own right, with a superimposed epileptic encephalopathy further adversely affecting development and cognition. Recent genomic advances, especially in DNA sequencing, have identified an increasing number of variations in genes known to cause DEEs, particularly those encoding ion channels and neurotransmitter receptors (e.g., *SCN2A, KCNA2*, *KCNB1*, *KCNQ2*, *KCNT1*, *KCNT2,* and *STXBP1)* (Wild and Nelson, 2019; Kessi et al., 2020; Kim et al., 2020).

Potassium (K^+^) channels, the most diverse ion channel group, are vital for neuronal excitability and signaling. There are five types of K^+^ channels categorized based on the stimulus that activates them: voltage-gated (KV), calcium-activated (K_Ca_), inwardly rectifying (Kir), ATP-sensitive (K_ATP_), and sodium-activated (K_Na_) potassium channel sub-families (González et al., 2012; Li et al., 2018). In humans, two K_Na_ channels have been described, Slack (also called Slo2.2) and Slick (also called Slo2.1), encoded by the *KCNT1* gene (OMIM* 608167) and *KCNT2* gene (OMIM* 610044), respectively. These channels are widely expressed in the central nervous system and are crucial in modulating membrane hyperpolarization resulting from repetitive firing and hetero-tetrameric channel formation in distinct brain regions (Chen et al., 2009; Rizzi et al., 2015).

The first reported heterozygous germline variant in the KCNT2 gene appeared in 2017 in a 4-year-old male with epileptic encephalopathy, characterized by neonatal hypotonia, intractable infantile-onset epilepsy, and profound DD (Gururaj et al., 2017). Recent studies have discovered pathogenic variants in the *KCNT2* gene, leading to developmental and epileptic encephalopathy-57 (DEE57; MIM 617771) in 25 patients (Gururaj et al., 2017; Ambrosino et al., 2018; Alagoz et al., 2020; Inuzuka et al., 2020; Mao et al., 2020; Gong et al., 2021; Jackson et al., 2021; Cioclu et al., 2023). Advances in genomics, especially next-generation sequencing, have increasingly linked DEEs to genetic variations (Zhou et al., 2018). Treatment for DEEs focuses on symptomatic management through antiepileptic drugs and rehabilitation for DD. Recent studies show significant advancements in the treatment of DEEs. New therapeutic options, including precision therapies and repurposed drugs, are emerging, potentially improving seizure burden and neurological outcomes (Vasquez et al., 2022). Among them, these new medications include cannabidiol, everolimus, and repurposed drugs like fenfluramine, currently being used for the management of DEEs (Johannessen Landmark et al., 2021). Despite advances in treatment, the long-term prognosis of DEE is influenced by various factors, including the type of seizures, underlying causes, severity, and response to treatment, with many patients facing challenges, especially in their response to multiple Antiseizure Medications (ASMs) (Bravo et al., 2021; Samanta, 2021; Kienitz et al., 2022). Here, we reported a novel *KCNT2* missense variant discovered in a family affected by developmental and epileptic encephalopathies.

## Case description

**FIGURE 1:**
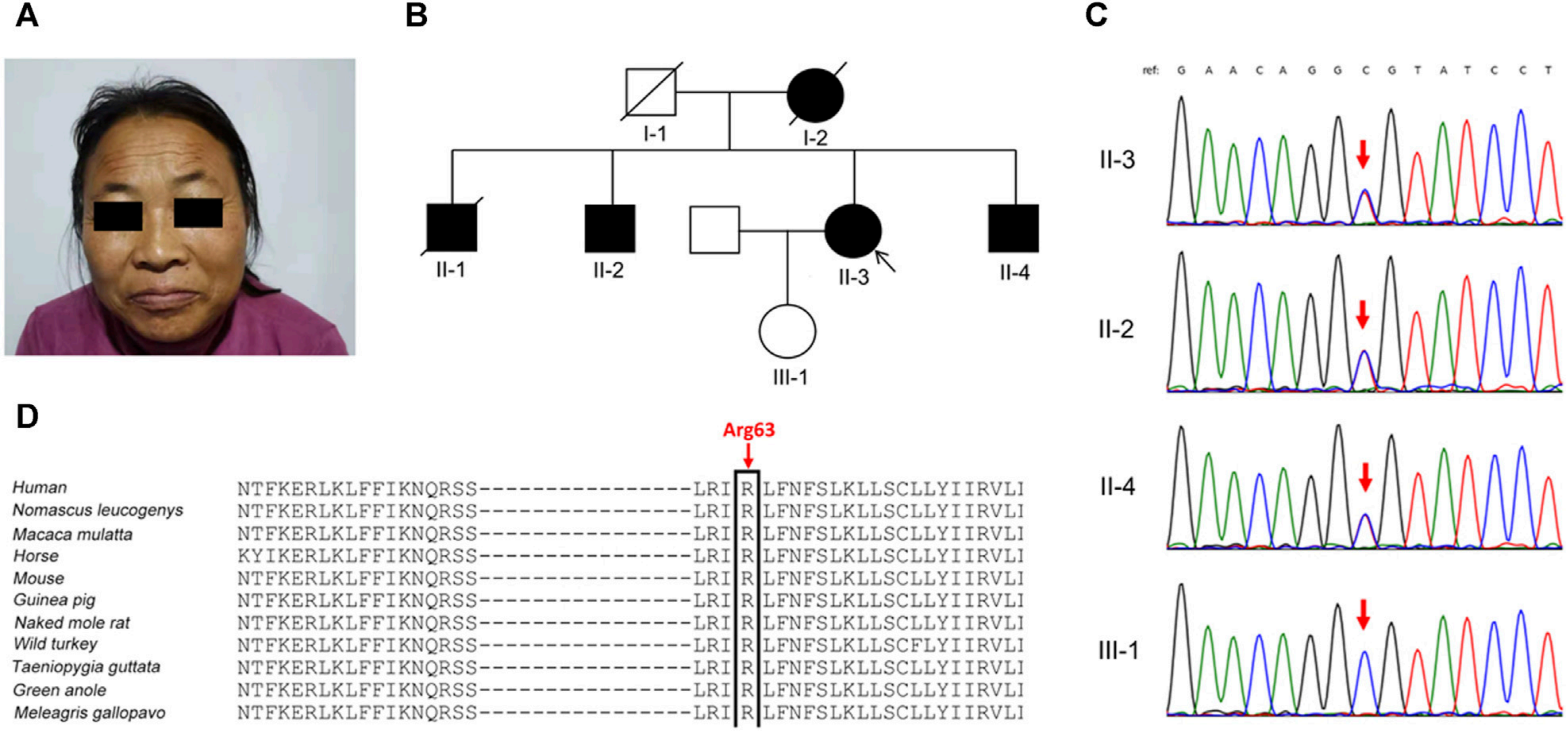
Clinical phenotypes and genetic analysis in the family. **(A)** The proband (Ⅱ-3) presented with prominent eyebrows, thick hair, and coarse facial features. **(B)** Pedigree of the family. **(C)** Sanger sequencing confirmed the *KCNT2* variant c.188G>A (p.Arg63His) in the proband and her two brothers (Ⅱ-2 and Ⅱ-4), and not in her clinically normal daughter (red arrow). **(D)** The Arg63 residue is highly evolutionarily conserved among different species.

The proband (Figure 1A), a 46-year-old woman of Han Chinese ethnicity, presented with early-onset epilepsy and ID, along with dysmorphic facial features (hypertrichosis and coarse facial features), and short stature (149.0 cm, below the third centile). A medical genetics outpatient clinic was consulted after her healthy 28-year-old daughter (Ⅲ-1) was advised to consider genetic testing before pregnancy due to the positive family history. The proband experienced initial epileptic seizures in infancy and occasional seizures during her childhood and adulthood, which were classified as focal to bilateral tonic-clonic seizures according to the 2017 ILAE seizure type classification. She had unpredictable seizure frequency, and the seizures usually resolved spontaneously within minutes. The latest seizure, triggered by emotional upset a year ago, involved limb stiffness and transient aphasia without loss of consciousness. The proband did not undergo brain MRI due to financial and geographical limitations. However, she had a normal head computed tomography (CT) scan, which excluded any major structural abnormalities. She had mild hypertrichosis and coarse facial features, such as thick hair and eyebrows, mildly downward-slanting eye corners, a broad nasal bridge, a relatively long philtrum, full and prominent lips, a wide mouth, and a slightly small lower jaw. Intellectually, she demonstrated lower cognitive function, with clear but slightly slow speech and a regular voice tone. She showed a limited capacity for deep thought and calculations. Nevertheless, she did not have any other comorbidities, such as cardiac, renal, or endocrine disorders. She also did not have any behavioral or psychiatric problems, such as autism spectrum disorder, attention deficit hyperactivity disorder and so on. Up to now, she has never received treatment with antiepileptic drugs.

In the proband’s pedigree (Figure 1B), similar clinical phenotypes were observed in two elder brothers (Ⅱ-1 and Ⅱ-2), a younger brother (Ⅱ-4), and their mother (Ⅰ-2), who had passed away. The eldest brother (Ⅱ-1) had the most frequent epileptic seizures and died at 42 years old. The second eldest brother (Ⅱ-2) experienced fewer epileptic-like episodes, characterized by unclear consciousness, limb stiffness, and speech impairment, resembling a “shock” state during the most severe episodes. The youngest brother has no history of epilepsy but has an ID. Both the proband’s husband and daughter were clinically normal, and her daughter’s height was 165 cm.

Diagnostic clinical exome sequencing revealed a novel heterozygous missense variant in the *KCNT2* gene (NM_198503: c.188G>A, p.Arg63His) in the proband and her two affected brothers, while her daughter did not carry this variant. Sanger sequencing confirmed the variant (Figure 1C). The variant p.Arg63His, found at a low frequency (2 out of 230,620 alleles) in the gnomAD database, is located at the junction of the N-terminal domain and S1 domain, showing high evolutionary conservation among different species (Figure 1D). The variant p.Arg63His was predicted to be damaging by various algorithms, including SIFT, Polyphen-2, MutationTaster, PROVEAN, and CADD. Based on these clinical and genetic characteristics, the affected individuals in this family were diagnosed with DEE caused by the *KCNT2* variant.

## Discussion

**FIGURE 2:**
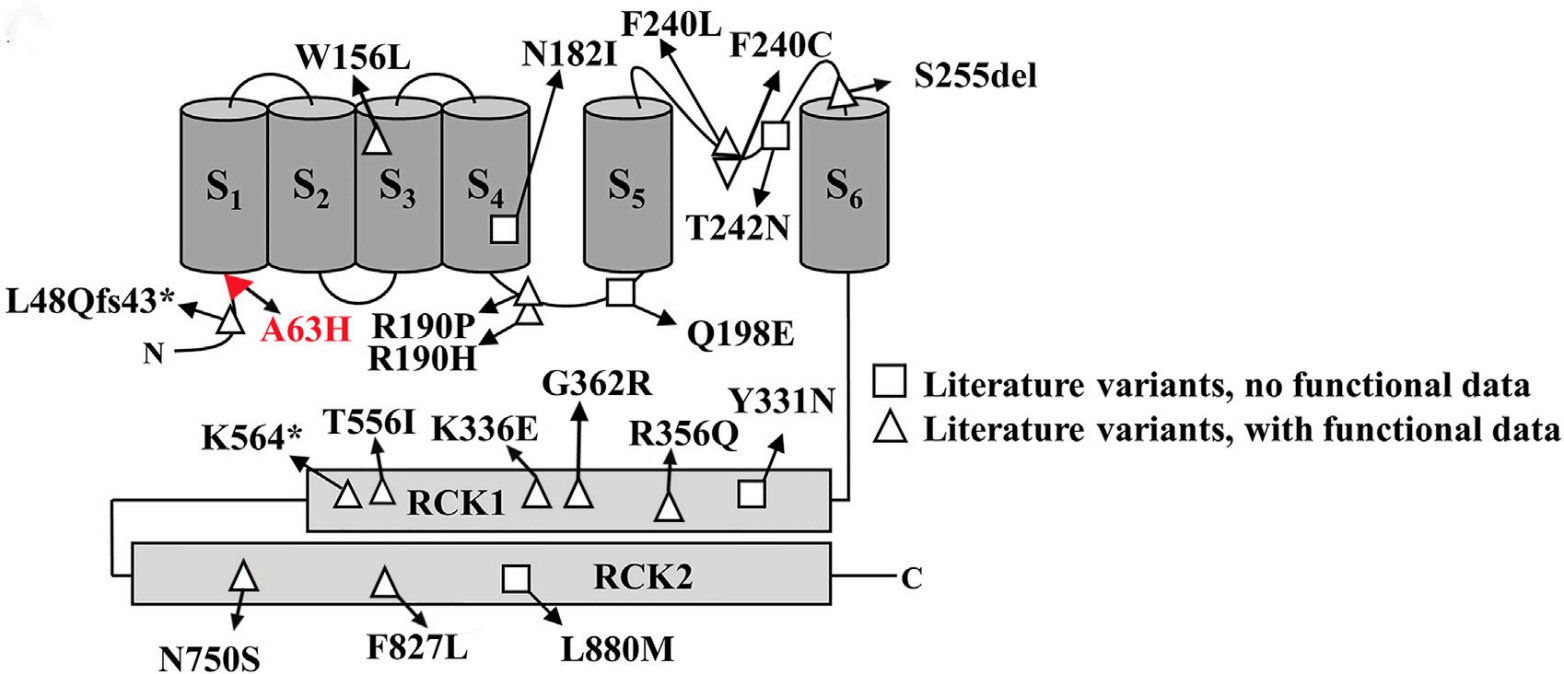
Schematic representative of the Slick channel with all published *KCNT2* variations. The position of the variant Arg63His is located at the junction of the S1 domain and N-terminal domain, which is indicated in red.

In this study, we reported a novel *KCNT2* gene variation (p.Arg63His) in a family and provided a comprehensive review of available literature on *KCNT2* variations (Table 1) (Gururaj et al., 2017; Ambrosino et al., 2018; Alagoz et al., 2020; Inuzuka et al., 2020; Mao et al., 2020; Gong et al., 2021; Jackson et al., 2021; Cioclu et al., 2023). Among the 25 patients previously reported, 19 pathogenic *KCNT2* variants were identified, including 16 missense variants, 1 in-frame deletion, 1 nonsense variant, and 1 frameshift variant. The only recurrent variants were observed at p190 position (R190H in patients #2, #3, #15, #16, #17; R190P in patients #18 and #19) (Ambrosino et al., 2018; Jackson et al., 2021; Álvarez-Mora et al., 2022). The location of each variant in the *KCNT2* subunit is shown in Figure 2. According to the clinical phenotypes described in the literature, patients with *KCNT2*-relevant diseases usually present with early-onset epileptic seizures, intellectual impairment, infantile hypotonia, motor DD, dysmorphic features, and typical EEG. It is noteworthy that early onset epileptic seizures, ID/DD, infantile hypotonia, dysmorphic features, and typical EEG were reported in 14, 19, 13, 13, and 16 of the patients, respectively. Meanwhile, Ambrosino et al. (Ambrosino et al., 2018) reported missense *KCNT2* variants (p.Arg190His and p.Arg190Pro) in two individuals, and they were both presented with epilepsy, intellectual disability, hypertrichosis, abnormal facial features, and short stature. However, Jackson et al., 2021 described two patients with the same variants, p.Arg190His and p.Arg190Pro, who had similar clinical phenotypes as described above but with no epilepsy. In this study, the proband and her four family members presented with similar clinical symptoms of dysmorphic features (hypertrichosis and coarse facial features), short stature, early-onset seizures, and intellectual disability.

**TABLE 1:** Overview of the phenotypic and genetic findings of all identified patients with *KCNT2*-relevant diseases (*n* = 26).

Pts	Genotype	Funct	Sex	Age	Age of epilepsy onset	Epilepsy	ID/DD	Neuro feat	Hypert	Coarse facial features	Height	Neuroradiology (type, age performed)	EEG	Treatm	Sz outcome
**Pt 1** This paper	c.188G>A (p.Arg63His)	N.A.	F	46 years	infantile	yes	mild	no	yes	thick eyebr., mildly downward-slanting eye corners, broad nasal bridge, long philtr., full and prominent lips, wide mouth, slightly small lower jaw	46years: 150 cm, short stature	CT (Normal), MRI (N.A.)	N.A.	no ASM	sz free
**Pt 2** Maria Cristina Cioclu et al. (2023) (pt#1)	c.467G>T (p.W156L)	LoF	M	10 years	14 m	yes	mild	Bab., weakn lower limbs, paraso	no	no	N.A.	MRI (Normal)	bi-Fr SW	VPA	sz free (10 years)
**Pt 3** Maria Cristina Cioclu et al. (2023) (pt#2)	c.569G>A (p.R190H)	GoF	F	5 years	—	no	mod	hyp, parox. dyst., arouse	yes	diast., curved eyebr., long eyelas, short philtr., full lips	N.A.	MRI (mild pachygyria)	Normal	—	—
**Pt 4** Maria Cristina Cioclu et al. (2023) (pt#3)	c.569G>A (p.R190H)	GoF	M	6 years	6 m	yes	severe	mild hyp	yes	arge eyebr., long eyelas., brachy	N.A.	MRI (mild volume loss)	hyps, poly-SW	no ASM	sz free (1year 1m)
**Pt 5** Maria Cristina Cioclu et al. (2023) (pt#4)	c.719T>G (p.F240C)	GoF	F	13.5 years	1 m	yes	mod-severe	no	yes	round nasal tip, short philtr., full lips, thick eyebr, long eyelas, hypert	N.A.	MRI (Normal)	hyps, poly-SW	no ASM	sz free (4 m)
**Pt 6** Maria Cristina Cioclu et al. (2023) (pt#5)	c.763_765del (p.S255del)	LoF	F	10 years	—	no	mod	no	no	↑IOD, flat nasal root, round tip, brachy, clino, synd	N.A.	MRI (Normal)	N.A.	—	—
**Pt 7** Maria Cristina Cioclu et al. (2023) (pt#6)	c.763_765del (p.S255del)	LoF	F	40 years	—	no	mild	no	no	no	N.A.	N.A.	N.A.	—	—
**Pt 8** Maria Cristina Cioclu et al. (2023) (pt#7)	c.1067G>A (p.R356Q)	GoF	F	22 m	4.5 m	yes	not appl	hyp	no	no	N.A.	MRI (Normal)	hyps, now Normal	KD, CBD, Quinid	sz free (9 m)
**Pt 9** Maria Cristina Cioclu et al. (2023) (pt#8)	c.1084G>C (p.G362R)	LoF	F	5 years	—	no	no	no	no	no	N.A.	N.A.	N.A.	—	—
**Pt 10** Maria Cristina Cioclu et al. (2023) (pt#9)	c.1096A>G (p.K366E)	GoF	M	6 years	—	no	mod-severe	hyp, weakn lower limbs, gait ataxia, arouse	no	no	N.A.	MRI (Normal)	Sh-W FrC > R	—	—
**Pt 11** Maria Cristina Cioclu et al. (2023) (pt#10)	c.1667C>T (p.T556I)	LoF	M	6 years 5 m	—	no	mod	ataxia clums, poor motor skills	no	long eyelas, short philtr, full upper lip, tooth shift	N.A.	N.A.	multiF Sh-W	—	—
**Pt 12** Maria Cristina Cioclu et al. (2023) (pt#11)	c.2249A>G (p.N750S)	GoF	M	14 years	6 m	yes	severe	hyp, ataxia	no	no	N.A.	MRI (Normal)	F S CPT-L	Sulth., KD	sz free (10 years)
**Pt 13** Maria Cristina Cioclu et al. (2023) (pt#12)	c.2479T>C (p.F827L)	GoF	M	22 years	—	no	mod	no	no	ear malf., campt, clino	N.A.	N.A.	N.A.	—	—
**Pt 14** Mao et al. (2020) (ptB)	c.143_144delTA (L48Qfs43*)	LoF	F	29 years	4 m	yes	Delayed	N.A.	N.A.	N.A.	N.A.	N.A.	N.A.	N.A.	N.A.
**Pt 15** Mao et al. (2020) (ptB)	c.545A>T (N182I)	LoF	M	6 years	N.A.	yes	Profound	Hyp, unable to walk	N.A.	N.A.	N.A.	MRI (thin CC, dilat lat. Vn.)	Sh-W and slow Fr-T R	N.A.	DR
**Pt 16** Ambrosino et al. (2018) (pt#1)	c.569G>A (R190H)	GoF	F	9 years	8 m	yes	Severe	Hyp	yes	Prominent eyebr, long eyelas, short philtr., diast	9years: 122 cm (−2.20 SDS)	MRI (Atrophy, delayed myel.)	Hyps - > Sh-slow-W, gen slow - > bil S	Sulth, VPA, VGB, TPM, LEV, CLB, GBP,LTG,RFM, MP, KD,Quin	DR
**Pt 17** Jackson et al. (2021) (pt#2)	c.569G>A (R190H)	GoF	F	5,5 years	—	no	Moderate	hyp, sleep disorder, falls	yes	prominent eyebr, long lashes, spaced teeth	4years: 108 cm (+0.57 SD)	MRI (normal)	N.A.	—	—
**Pt 18** Jackson et al. (2021) (pt#29)	c.569G>A (R190H)	GoF	M	6 years	—	no	Severe	N.A.	no	elongated face, broadbasednose, short filter, prognathism	N.A.	N.A.	bil T and F epi	—	—
**Pt 19** Ambrosino et al. (2018) (pt#2)	c.569G>C (R190P)	GoF	F	13 years	1 day	yes	Severe	Hyp	yes	Prominent eyebr, long eyelas, short philtr., diast	12½y: 143.5 cm (−1.78 SDS)	MRI (normal)	Gen/multiF epi; sz migr	PB - now suspended	no ASMs
**Pt 20** Jackson et al. (2021) (pt#1)	c.569G>C (R190P)	GoF	F	32 years	—	no	Severe	Hyp	yes	synophrys, long eyelas, diast	14years: 170 cm (+1.46 SD)	CT (normal)	N.A.	—	—
**Pt 21** Gong et al. (2021) (pt#2)	c.592C>G (Q198E)	GoF	M	9 years	1.5 m	yes	Profound	regression, imp walking	on	Prominent eyebr, long eyelas, short philtr., hirsutism	8years: 118cm, short stature	MRI (Normal)	MultiF epi, gen epi	VPA, TPM, LTG, NZP	DR
**Pt 22** Gururaj et al. (2017)	c.720T>A (F240L)	change*	M	10 years	3 m	yes	Profound	Hyp, poor VA, imp. walking, insomnia	no	no	Normal	MRI (↓WM, thin CC)	multiF epi, hyps	TPM, NRZ, LEV, LTG, VGB, ESM, ZNS, VPA, KD, UKISS	DR
**Pt 23** Inuzuka et al. (2020)	c.725C > A (T242N)	LoF	M	17 years	5 m	yes	Severe	Spasticity, ataxia	N.A.	N.A.	N.A.	MRI (normal)	multiF epi; sz onset ant. bil	CBZ, LEV and OXC, other ASMs	DR
**Pt 24** Gong et al. (2021) (pt#1)	c.991T>A (Y331N)	GoF	M	5 m	8 days	yes	Not applic	hyp, poor VA	no	Prominent eyebr, long eyelas, short philtr., hirsutism	N.A.	MRI (normal)	burst suppression - > hyps	PB, TPM	DR
**Pt 25** Mao et al. (2020) (pt A)	c.1690A>T (K564*)	LoF	F	3 m	2 m	yes	Not applic	Hyp, limited VA	N.A.	N.A.	N.A.	MRI (normal)	slow bg, multiF epi; sz migrating	VPA, LTG and LEV; other ASMs	DR
**Pt 26** Alagoz et al. (2020) (pt#2)	c.2638C>A (L880M)	GoF	M	5 years	N.A.	yes	Delayed	N.A.	N.A.	N.A.	N.A.	N.A.	normal	N.A.	DR

ASM: anti-seizure medication, appl: applicable, Bi-Fr: bilateral frontal, CBD: cannabidiol, CBZ: carbamazepine, CLB: clobazam, *Change of function: loss of K+ specificity and permeability to other cations, d: days, DD: developmental delay, diast: diastema, DR: drug-resistant, eyelas: eyelashes, eyebr: eyebrows, ESM: ethosuximide, F: female, Feat: feature, F: focal, Fr: frontal, Funct: functional properties, GBP: gabapentin, GoF: gain of function, hyp: hypotonia, hypert: hypertrichosis, hyps: hypsarrhythmia, ID: intellectual disability; ↑IOD: increased intra-ocular distance (hypertelorism), KD: ketogenic diet, L: left, multiF: multifocal, LEV: levetiracetam, LoF: loss of function, LTG: lamotrigine, M: male, mod: moderate, MP: methylprednisolone, m: months, multiF: multifocal, N.A.not available, Neuro: neurological, NRZ: nitrazepam, OXC: oxacarbazepine, PB: phenobarbital, P: parietal, Pt: Patient, philtr: philtrum, Quin: quindine, RFM: rufinamide, Sz: seizure, Sulth: Sulthiame, S: spikes, Sh-W: sharp wave, SW: spike and wave, T: temporal, TPM: topiramate, Treatm: treatment, VA: visual attention, VGB: vigabatrin, VPA: valproate, ZNS: Zonisamide. y: years.

Potassium channel dysfunction caused by genetic variations can lead to various neurological disorders. These disorders have clinical manifestations, including multiple forms of epilepsy, ID, and autism, among other conditions (Huang et al., 2018; Garrido, 2023). In the present study, five Chinese family members reported similar clinical symptoms of early-onset seizures, intellectual disability, hypertrichosis, coarse facial features, and short stature. Clinical exome sequencing revealed a novel heterozygous variant in the *KCNT2* gene (c.188G>A, p.Arg63His) in the proband and her two affected brothers, while her healthy daughter did not carry this variant. We identified the p.Arg63His variant as the disease-causing variant in this family.

Numerous studies have shown that K_Na_ channels contribute to slow afterhyperpolarization, the adaption of firing frequency in neurons, and the stabilization of resting membrane potential (Gao et al., 2008; Kaczmarek, 2013). Two known genes, *KCNT*1 and *KCNT2*, encode K_Na_ channel subunits Slack and Slick. Slack and Slick’s channels contain six membrane-spanning domains (S1-S6), an intracellular N-terminus, and a long C-terminus (Kaczmarek, 2013). Slick’s amino acid sequence shows high homology with Slack’s, but the amino-terminal sequences display a notable difference. Previous functional studies of *KCNT2* variants have shown that 10 variants (R190H, F240C, R356Q, K366E, N750S, F827L, R190P, Q198E, Y331N, L880M) exhibit gain-of-function (GoF) characteristics. In comparison, 8 variants (W156L, S255del, G362R, T556I, L48Qfs43*, N182I, T242N, K564*) show loss-of-function (LoF) phenotypic features (Gururaj et al., 2017; Ambrosino et al., 2018; Alagoz et al., 2020; Inuzuka et al., 2020; Mao et al., 2020; Gong et al., 2021; Jackson et al., 2021; Cioclu et al., 2023). Therefore, although the *KCNT1* and *KCNT2* channels are structurally and functionally similar, they seem to exhibit significant differences in the functional characteristics of pathogenic variants causing DEEs. In in vitro evaluations, *KCNT1* variants almost always promote a GoF phenotype, whereas *KCNT2* variants are almost evenly distributed between GoF and LoF (10 and 8, respectively). Moreover, the Slick channel uniquely regulates neuronal excitability, characterized by its rapid gating kinetics and sensitivity to intracellular ATP levels, in contrast to the Slack channel, which has been identified with potential sensitivity to intracellular chloride and sodium ions (Bhattacharjee et al., 2003; Xu et al., 2023). Research indicates that the mammalian central nervous system widely but heterogeneously distributes Slack and Slick’s channels, which are co-expressed by many types of central neurons (Rizzi et al., 2015; Rizzi et al., 2016). Within the dorsal root ganglia (DRGs), knockout of *KCNT1* abolishes K_Na_ current, and *KCNT1* knockout mice exhibit enhanced itch and pain responses. However, the knockout of *KCNT2* in the DRGs does not cancel K_Na_ current (Martinez-Espinosa et al., 2015). A study from *KCNT2* knockout mice revealed that Slick channels inhibit the excitability of calcitonin gene-related peptide (CGRP)-containing neurons, relieving pain after inflammation and injury (Tomasello et al., 2017). Until now, we still know very little about the clearly defined physiological function of the *KCNT2* gene.

In 2017, Gururaj et al., 2017 first reported a heterozygous *KCNT2* variation (Phe240Leu) in a patient with an early onset epileptic encephalopathy (EOEE), and relevant experiments confirmed a “change-of-function” effect of the variation by altering ion selectivity. The p.Phe190 residue of Slick is situated between helices S4 and S5, and variations in the *KCNT2* gene affecting the Phe190 residue have been reported in four patients with DEE and dysmorphic features (Ambrosino et al., 2018; Jackson et al., 2021). Experimental results showed that Arg190His and Arg190Pro increase maximal K^+^ current densities and shift toward more negative membrane potentials, consistent with GoF effects (Ambrosino et al., 2018). Scientists have discovered two truncating alterations in *KCNT2* (p.Leu48Glnfs43, p.Lys564). These alterations, predicted to be null variants, reduce the global current density of heteromeric channels, thereby impacting K_Na_ function (Mao et al., 2020). Nonetheless, the harmful mechanism of truncating alterations is not due to haploinsufficiency, as the *KCNT2* gene is likely to tolerate LoF alterations more (PLI = 0.04). Further research, including functional studies, is required to elucidate the pathogenic mechanism of the *KCNT2* variant p.Arg63His in this study. Only a few studies have investigated patients with DEE due to *KCNT2* variation, and the number of functional analyses performed is also limited. Thus, the establishment of genotype-phenotype correlations still needs to be completed.

Hypertrichosis and coarse facial features have been reported in several K^+^ channel opathies caused by variations in the *KCNH1*, *KCNN3*, *KCNK4*, and *KCNJ8* genes (Cooper et al., 2014; Gripp et al., 2021; Apuril Velgara et al., 2022). Notably, K_ATP_ channels are uniquely evolved protein complexes that couple intracellular metabolism to the electrical activity by regulating plasma membrane K^+^ flux in response to changes in the intracellular concentrations of ATP and ADP, thus playing an essential role in the process physiological and pathophysiology (Driggers and Shyng, 2021). K_ATP_ channels are composed of an inwardly rectifying K^+^ channel subunit, either Kir6.1 (*KCNJ8* gene) or Kir6.2 (*KCNJ11* gene), plus a sulfonylurea receptor, either SUR1 (*ABCC8* gene) or SUR2 (*ABCC9* gene) that serve as the regulatory subunit. Research reports indicate that variations in *KCNJ8* or *ABCC9* cause Cantú syndrome. This syndrome features congenital hypertrichosis, distinctive facial characteristics such as a broad nasal bridge, long philtrum, a wide mouth with prominent lips, osteochondrodysplasia, and cardiovascular abnormalities. Studies demonstrate that pathogenic variants in *ABCC9* or *KCNJ8* increase the opening of the K_ATP_ channel resulting from decreased ATP-mediated inhibition, consistent with gain-of-function variations (Harakalova et al., 2012; McClenaghan et al., 2018). Minoxidil and diazoxide, which are K_ATP_ channel agonists, were initially used as antihypertensive drugs and commonly resulted in the side effects of hair overgrowth (Newfield, 2015; Suchonwanit et al., 2019).

Previous studies have shown that the Slick channel functions as a hybrid between two classes of K^+^ channels, named K_Na_ channels and K_ATP_ channels (Bhattacharjee et al., 2003). The Slick channel can be activated via intracellular Na^+^ and Cl^−^ and inhibited by intracellular ATP. Patients with *KCNT1* variations have not reported hypertrichosis and coarse facial features. The clinical phenotypes of *KCNT2* variations largely overlap with K_ATP_ channel diseases, which include epilepsy, ID/DD, hypertrichosis, and coarse facial features. Besides, patients carrying GoF *KCNT2* variants often presented with more severe ID/DD, earlier epilepsy onset, and pronounced dysmorphisms, including hypertrichosis, compared to those with LoF variants (Cioclu et al., 2023). Hypertrichosis occurs in patients with K^+^ channelopathies that might be partial, local, or distributed over the whole body (Brownstein et al., 2013; Gripp et al., 2021). In this study, the proband presented with mild hypertrichosis characterized by thick scalp hair and prominent eyebrows, which improved gradually with age. This is consistent with the findings in the literature, where GoF variants were more frequently associated with severe ID/DD and earlier epilepsy onset (Cioclu et al., 2023). Short stature has been reported in three patients with *KCNT2* variations (Ambrosino et al., 2018; Gong et al., 2021). In this study, all affected patients presented with short stature. However, the underlying mechanism of short stature by *KCNT2* variations is unknown.

In our study, the proband is currently seizure-free and has no ASM treatment. While GoF *KCNT2* variants are universally blocked by quinidine and fluoxetine, LoF variants like W156L or N182I exhibit a different pharmacological profile, being potentiated by loxapine or riluzole (Gururaj et al., 2017; Ambrosino et al., 2018; Alagoz et al., 2020; Inuzuka et al., 2020; Mao et al., 2020; Gong et al., 2021; Jackson et al., 2021; Cioclu et al., 2023). This implies that the same drug can have varying effects depending on the specific *KCNT2* variant, necessitating more tailored pharmacological interventions based on the particular *KCNT2* variant present in each patient.

In conclusion, our study, in light of recent findings, confirms the diverse clinical spectrum of *KCNT2*-related disorders and underscores the importance of comprehensive genetic and clinical evaluations for accurate diagnosis and management. However, our study faces limitations, including the inability to perform genetic testing on all key family members and the lack of comprehensive neuropsychiatric evaluations due to socioeconomic and geographical challenges. Moreover, establishing a clear genotype-phenotype correlation for *KCNT2*-related disorders requires more extensive studies and functional analyses of various *KCNT2* variants.

## Conclusion

In this study, we report a novel *KCNT2* variant (p.Arg63His) in a family and comprehensively review available literature concerning *KCNT2* variations. The *KCNT2* variants can be classified into GoF or LoF, depending on their effects on channel current. Epilepsy or ID/DD occurs in almost all patients with *KCNT2* variations, while hypertrichosis and distinctive coarse facial features are also commonly found. We observed that the GoF variants are associated with more severe epilepsy and DD, which may be due to an increase in channel activity and neuronal excitability, leading to hyperexcitability and seizures. Conversely, LoF variants are associated with milder epilepsy and variable developmental outcomes. The clinical phenotypes of *KCNT2*-relevant DEEs partially overlapped with *KCNT1* variations and K_ATP_ channel diseases, mainly due to the hybrid function between K_Na_ and K_ATP_ channels. We speculate that patients with GoF *KCNT2* variations usually present with epilepsy, ID/DD, hypertrichosis, and coarse facial features; LoF *KCNT2* variations are unlikely to lead to hypertrichosis and coarse facial features. However, further research and functional studies are necessary to make this conclusion. In conclusion, our study contributes to the evolving understanding of *KCNT2*-related disorders, highlighting the significance of GoF and LoF variants in determining the severity and range of clinical phenotypes, including ID/DD and hypertrichosis. This insight is crucial for tailoring appropriate therapeutic interventions and future research in this field.

## Data Availability

The original contributions presented in the study are included in the article/Supplementary material, further inquiries can be directed to the corresponding author.
